# Effect of treatment with growth hormone on body composition and metabolic profile of short children born small for gestational age

**DOI:** 10.1590/1984-0462/2024/42/2023073

**Published:** 2024-02-12

**Authors:** Adriana Masiero Kühl, Márcia Regina Messaggi Gomes Dias, Rosana Marques Pereira

**Affiliations:** a Universidade Estadual do Centro-Oeste, Guarapuava, PR, Brazil.; b Universidade Federal do Paraná, Curitiba, PR, Brazil.

**Keywords:** Infant, small for gestational age, Body composition, Recombinant growth hormone, Recém-nascido pequeno para a idade gestacional, Composição corporal, Hormônio do crescimento recombinante

## Abstract

**Objective:**

To assess the effect of recombinant growth hormone (rGH) on body composition and metabolic profile of prepubertal short children born small for gestational age (SGA) before and after 18 months of treatment.

**Methods:**

It is a clinical, non-randomized, and paired study. Children born SGA, with birth weight and/or length <-2 standard deviations (SD) for gestational age and sex, prepubertal, born at full term, of both genders, with the indication for treatment with rGH were included. The intervention was performed with biosynthetic rGH at doses ranging from 0.03 to 0.05 mg/kg/day, administered subcutaneously, once a day at bedtime. Total lean mass (LM) and total fat mass (FM) were carried out using dual-energy X-ray absorptiometry (DXA), and the metabolic profile was assessed for insulin, glycemia, IGF-1 levels and lipid profile.

**Results:**

Twelve patients (nine girls, 8.17±2.39 y) were evaluated; three patients dropped out of the study. There was an increase of LM adjusted for length (LMI) (p=0.008), LMI standard deviation score (SDS) adjusted for age and sex (p=0.007), and total LM (p<0.001). The percentage of body fat (BF%) and abdominal fat (AF) remained unaltered in relation to the beginning of treatment. Among the metabolic variables, blood glucose remained within normal levels, and there was a reduction in the number of participants with altered cholesterol (p=0.023).

**Conclusions:**

The effect of rGH treatment was higher on LM than in FM, with increased LM adjusted for length and standardized for age and sex. Glycemia remained within the normal limits, and there was a decreased number of children with total cholesterol above the recommended levels.

## RESUMO

**Objetivo:**

Avaliar o efeito do hormônio de crescimento recombinante (rHC) na composição corporal e no perfil metabólico de crianças pré-púberes com baixa estatura, nascidas pequenas para a idade gestacional (PIG) antes e depois de 18 meses de tratamento.

**Métodos:**

Estudo clínico, não randomizado e pareado. Foram incluídas crianças nascidas PIG, com peso e/ou altura ao nascer <-2 desvios padrão (DP) para idade gestacional e sexo, pré-púberes, nascidas a termo, de ambos os sexos, com indicação de tratamento com rGH. A intervenção foi realizada com rGH biossintético com doses variando de 0,03 a 0,05 mg/kg/dia, administrado por via subcutânea, uma vez ao dia ao deitar-se. A massa magra total (LM) e a massa gorda total (MG) foram determinadas por meio de absorciometria de raios X de dupla energia (DXA), e o perfil metabólico foi avaliado com dosagens de insulina, glicemia, IGF-1 e perfil lipídico.

**Resultados:**

Doze pacientes (nove meninas, 8,17±2,39 anos) foram avaliados; três pacientes abandonaram o estudo. Houve aumento da LM ajustada para estatura (LMI) (p=0,008), LMI *standard deviation scores* (SDS) ajustada para idade e sexo (p=0,007) e LM total (p<0,001). O percentual de gordura corporal (GC%) e gordura abdominal (AF) permaneceu inalterado em relação ao início do tratamento. Entre as variáveis metabólicas, a glicemia manteve-se na normalidade, e houve redução do número de participantes com colesterol alterado (p=0,023).

**Conclusões:**

O efeito do tratamento com HCr foi maior na MM do que na MG, com o aumento da MM ajustada para altura e padronizada para idade e sexo. A glicemia permaneceu normal e houve redução do número de crianças com colesterol total acima do recomendado.

## INTRODUCTION

Childhood is a period of fast growth and development, highly vulnerable to health complications in the presence of unfavorable conditions. Newborns with weight and/or length below -2 standard deviation scores (SDS) for gestational age and sex are classified as small for the gestational age (SGA) and are more susceptible to health problems in the neonatal period and development of chronic diseases in adulthood. The causes for SGA are diverse and can be associated with intrauterine growth restriction, ethnicity, or short maternal stature.^1–3^

Most children born SGA show spontaneous catch-up growth into normal length range (>-2 SDS) during childhood, but about 10% remain short after four years old and exhibit less lean mass (LM). In turn, when there is a fast and increased body weight during the catch-up growth period, these children may exhibit higher fat mass (FM), with deposition of fat in the central region of the body, higher blood glucose and body pressure levels.^4,5^

Recombinant growth hormone (rGH) treatment has shown good tolerance, low incidence of adverse effects and positive results in increasing stature, in improving lipid metabolism and regularizing blood pressure and metabolic factors. rGH also changes the body composition of these children, contributing to increasing LM or maintaining or diminishing FM.^4,6^

This treatment is recommended for children born SGA with a length <-2 standard deviations (SD) and at least two years old. The treatment should preferably begin in prepuberty, with doses ranging from 0.03 to 0.07 mg/kg/day and can last until the children reach adult length.^7–13^

Body composition in most of these studies was assessed by dual-energy X-ray absorptiometry (DXA), which is considered the gold standard for assessment of body mass composition, especially FM. This method has good accuracy, is fast, painless, and the child can stay awake during the exam. The DXA execution method is safe, using low-intensity X-rays, which allows to measure bone mass, LM and FM individually.^14,15^

Some studies show a significant increase in LM throughout the treatment, at the end of the follow-up period, and in comparison with the control group, with the effect being more evident in boys and during the first year of treatment. The main determinant of reported LM gain is length, as growth recovery was accompanied by an increase in LM, especially during the first year of treatment. It’s noteworthy that children who underwent the treatment gained less FM than those who were untreated.^7,13,16,17^

Therefore, it can be assumed that children born SGA, when subjected to growth hormone therapy, experience an improvement in growth and body composition. This study aimed to assess the effect of rGH treatment on body composition and metabolic health of short children born SGA before and after 18 months of treatment.

## METHOD

It is a clinical, non-randomized, paired study carried out with short children born SGA who received treatment with rGH at the Pediatric Endocrinology Unit of the Clinical Hospital Complex at the Federal University of Paraná (UEP/CHC/UFPR) from October 2018 to December 2021.

Eligibility criteria included prepubertal short children of both sexes born SGA,^18^ with weight and/or length at birth <-2SD for gestational age and sex,^19^ born at full term, with indication for treatment with rGH and whose parents and/or guardians agreed to participate in the study and signed the Free and Informed Consent Form in writing.

Children born preterm, with bone dysplasia, genetic syndromes, deficiency of growth hormone and other hormones, and children already in puberty at the beginning of the treatment were excluded from the study.

Twelve patients were included in the study and evaluated in two moments: immediately before starting treatment with rGH and 18 months later. Three children were lost to follow-up, resulting in nine children evaluated at the end of the study. Losses during the study comprised individuals who abandoned treatment (n=1) and conclusion of data collection before completing 18 months of treatment (n=2).

All children were followed up by pediatric endocrinologists and received treatment with biosynthetic rGH with doses ranging from 0.03 to 0.05 mg/kg/day, administered subcutaneously, once a day at bedtime.

Body mass composition and metabolic variables were assessed prior to the beginning of the treatment (4.0±1.7 months before) and 18 months afterwards. All data were collected by one single researcher in both periods.

Body composition was assessed by Dual-Energy X-Ray Absorptiometry (DXA) at the Bone Densitometry Sector of the Clinical Hospital Complex of the Federal University of Paraná (CHC-UFPR), in a Lunar Prodigy Advance^®^ whole-body scanner (GE Medical Systems, Madison, WI, USA) and Encore^®^ software. All assessments were carried out using the same equipment, by the same person, with calibration performed every day in the morning prior to the first scan.

During the scans, the patients wore light clothes without metallic parts and were asked to remove all metallic objects. They were placed in supine position with the body centralized at the scanning table, with the feet in neutral position and secured with a Velcro strip, hands facing downwards, 1 cm away from the body, laying still as proposed by the NHANES’s protocol.^20^

Based on the DXA examination, FM values, percentage of body fat (BF%) and LM were determined. With absolute FM and LM values, the Lean Mass Index (LMI) [total lean mass (kg)/length (m^2^)] and Fat Mass Index (FMI) [total fat mass (kg)/length (m^2^)] were also calculated. All these values, FM, BF%, LM, LMI and FMI were transformed into Standard Deviation Scores (SDS) standardized for sex and age, based on two Brazilian reference populations, one for children under ten years of age^21^ and the other for those over one year.^22^

DXA also enables to assess regional distribution of body fat by using a standard configuration for segmental analysis, based on which the total abdominal fat (AF) and the percentage of abdominal fat (AF%) could be determined. This region, also called android region, comprises the area located between the ribs and pelvis, with demarcation greater than 20% of the distance between the iliac crest and the neck and a lower demarcation at the top of the pelvis.^14^

The clinical variables related to weight and length at birth, gestational age (GA) and pubertal stage were obtained based on a research protocol for data collection from medical records developed by UEP/CHC/UFPR. Current weight and length were measured using a digital scale (Filizola^®^) and fixed stadiometer (Stadiometer Mode S100^®^), respectively, and measurements were performed following the recommendations of the Ministry of Health.^23^ To assess nutritional status, the anthropometric indices of body mass index for age (BMI/A) and stature for age (S/A) were determined according to WHO’s recommendations and cutoff scores.^18^

The evaluation of the metabolic parameters was carried out at the Clinical Analysis Laboratory of the Clinical Hospital (ULAC/CHC/UFPR), with blood collected by vein puncture after 12 hours of fasting. Glucose, insulin, total cholesterol, HDL-cholesterol and triglycerides levels were measured using the Anility equipment, with specific kits for each component.

Lipid and insulin profiles were evaluated according to the *I Diretriz de Prevenção da Aterosclerose na Infância e na Adolescência* ^24^ [1^st^ Guideline for the Prevention of Atherosclerosis in Childhood and Adolescence], and the desirable levels considered were: for total cholesterol <150 mg/dL; for triglycerides and LDL-cholesterol (LDL-COL) <100 mg/dL; for HDL-cholesterol (HDL-COL) >45 mg/dL and insulin <15 mUI/L. For fasting glucose, a glucose level of <100 mg/dL was considered adequate.^25^

Insulin resistance (IR) was determined by the Homeostasis Model Assessment for Insulin Resistance (HOMA-IR) index using the HOMA2 Calculator^®^ software, which utilizes fasting glucose added by insulin in the formulation. HOMA-IR values over 2.5 units were defined.^26^

This study was approved by the Human Research Ethics Committee from CHC-UFPR with registration no. 94100318.4.0000.0096. All participants received the Informed Consent Form and, for those aged ten years or over, the Informed Assent Term was administered.

Data homogeneity was assessed using the Kolmogorov-Smirnov and Shapiro-Wilk normality tests, which showed normal distribution for most variables. Mean (X) and SD were used for descriptive statistics. For comparison of the means before and after 18 months of treatment, Student’s t-test was used for dependent samples, for variables with normal distribution. For variables that did not present normal distribution, the Wilcoxon test was used. The categorical variables were assessed by the McNemar test, and, when not possible, the Chi-Square test was used. Significance level was determined as p<0.05. The analyses were carried out using the Statistical Package for the Social Sciences (SPSS) statistical software (version 20).

The effect size was calculated by the Cohen’s d test, using the GPower 3.1.9.7 software, and values ≥0.8 were assumed as having large effect size; between 0.8 and 0.2 they were considered as medium effect size, and <0.2 as small effect size.

## RESULTS

Nine children participated in the study and were evaluated in pre-treatment period and after 18 months of treatment with rGH. All children were in the prepubertal period before the treatment, the initial mean age was 9.0±2.7, and final age was 10.7±2.7. The majority were female (55%), born at term (GA ≥38 weeks) and with length <-2 SD (Table 1). With respect to the anthropometric characteristics, only length (p<0.001) increased. Weight, BMI/A, and waist circumference stayed unaltered and within the normal levels (Table 2).

**Table 1:** Initial clinical characteristics of children born small for gestational age.

Variable	$\bar{\text{X}}\text{(SD)}$
GA (weeks)	38.9 (0.9)
Weight at birth (g)	2619 (525)
Weight at birth (z-score)	-1.4 (0.9)
Length at birth (cm)	44.1 (2.1)
Length at birth (z-score)	-2.6 (0.6)

GA: gestational age; SD: standard deviation

**Table 2:** Changes in the anthropometric characteristics from the beginning of the recombinant growth hormone treatment and after 18 months.

	Initial (n=9) $\bar{\text{X}}\text{(SD)}$	18 months (n=9) $\bar{\text{X}}\text{(SD)}$	p-value
Weight (z-score)	-1,6 (1,7)	-1,1 (1,0)	0,301
Stature (z-score)	-2.8 (0.4)	-1.8 (0.5)	<0.001
BMI (z-score)	-0.2 (1.2)	-0.1 (1.2)	0.537
Waist circumference (cm)	53.7 (6.9)	54.7 (14.7)	0.758

BMI: Body Mass Index; SD: standard deviation.

The results of assessment of body composition show that, after 18 months of treatment with rGH, there was an increase in LM (kg) from 17.1±5.9 kg to 22.7±7.5 kg (p<0.001), as well as in LM adjusted for length (LMI) and LMI SDS adjusted for age and sex. There was no change in FM when the same adjustments were used, as well as for BF%, AF and AF%, which remained unaltered (Table 3).

**Table 3:** Body composition at the beginning of the treatment with recombinant growth hormone and after 18 months of treatment.

	Initial (n=9) $\bar{\text{X}}\text{(SD)}$	18 months (n=9) $\bar{\text{X}}\text{(SD)}$	p-value	Variation	*d* *
LM (kg)	17.1 (5.9)	22.8 (7.6)	<0.001	5.62	0.81
LM (SDS)	-1.4 (1.4)	-0.6 (1.3)	0.162	–	–
LMI (Kg/m^2^)	12.3 (1.5)	13.1 (1.8)	0.008	0.78	0.46
LMI (SDS)	-0.4 (0.9)	0.2 (1.0)	0.007	0.56	0.58
BF%	17.6 (5.7)	19.5 (8.2)	0.382	–	–
BF% (SDS)	-0.6 (0.8)	-0.5 (0.9)	0.901	–	–
FM (kg)	3.8 (2.1)	6.1 (3.9)	0.033	2.34	0.69
FM (SDS)	-0.7 (0.7)	-0.6 (0.6)	0.426	–	–
FMI (kg/m^2^)	2.7 (1.2)	3.4 (1.9)	0.130†	–	–
FMI (SDS)	-0.4 (0.6)	-0.1 (0.9)	0.145	–	–
AF (kg)	0.3 (0.3)	0.4 (0.2)	0.960	–	–
AF%	17.0 (6.9)	19.8 (8.3)	0.181	–	–

SD: standard deviation; LM: total lean mass; LMI: lean mass index; SDS: standardized standard deviation scores; BF%: percentage of body fat; FM: total fat mass; FMI: fat mass index; AF: abdominal fat.

* Adjusted effect size (*Cohen’s D*)

† Teste de Wilcoxon

The treatment with rGH had better effect on LM than on the other variables (*d*=0.81), with an increase of 5.6 kg after 18 months of treatment (Table 3).

Table 4 contains the results of the metabolic evaluation, where it can be seen that the children started the treatment with total cholesterol levels above the recommended values (165.0±21.3 mg/dL), and there was an improvement after 18 months of treatment. An increase of IGF-1 levels (188.8±48.2 to 403.1±116.4 mg/ml) was found, and the glucose levels remained within the recommended levels for age.

**Table 4:** Metabolic profile at the beginning of the treatment with recombinant growth hormone and after 18 months of treatment.

	Initial (n=9) $\bar{\text{X}}\text{(SD)}$	18 months (n=9) $\bar{\text{X}}\text{(SD)}$	p-value
Insulin (mUI/dL)	5.7 (4.3)	10.3 (7.7)	0.139*
HOMA-IR (units)	0.7 (0.5)	1.3 (0.9)	0.139*
Glucose (mg/dL)	77.2 (6.5)	85.0 (6.9)	0.017
Total cholesterol (mg/dL)	165.0 (21.3)	158.3 (17.2)	0.324
LDL – cholesterol (mg/dL)	98.3 (18.0)	93.1 (16.4)	0.409
HDL – cholesterol (mg/dL)	50.9 (11.1)	47.2 (8.8)	0.336
Triglycerides (mg/dL)	72.4 (34.9)	99.8 (88.3)	0.508
IGF-1 (mg/ml)	188.8 (48.2)	403.1 (116.4)	0.001

SD: standard deviation

* Teste de Wilcoxon

With respect to the lipid profile, only total cholesterol exhibited a statistical difference between the assessments, showing a decrease in the number of participants with inadequate cholesterol levels from 75% (n=9) to 66.7% (n=6) (p=0.023).

## DISCUSSION

This study showed longitudinal results of 18 months of treatment with rGH in body composition assessed by DXA and in the metabolic profiles of short children born SGA. Children born SGA exhibited BMI/(z-score), LM (SDS), BF% (SDS) and FM (SDS) lower than the mean value for the population of the same age and sex at the beginning of the treatment, with an increase in total LM (kg), LMI and LMI SDS after the treatment period. There was an increase in the lipid profile, with a decreased number of children with altered cholesterol.

A study in the literature reports a profile similar to the one found here, where short-stature children born SGA usually exhibit lower BMI/A, combined with a lower amount of LM and FM, than children of the same age and sex born with adequate weight and/or stature for the gestational age.^7,10,11,13,16,27^ This condition may be the effect of a failure in catching-up growth in early childhood, when about 10% of the children born SGA remain smaller than other children of the same age and sex, causing children born SGA to have an average length score lower than the reference population.^28^

For these children, rGH treatment has been proven to be safe and with positive results both for length catch-up and for lipid profile and body composition, especially when this treatment starts during prepuberty.^4,6^ In this study, all children were at the prepubertal phase when the treatment began and succeeded in achieving catch-up in length, better levels of total cholesterol and increased LM.

LM increase was the major alteration found in the body composition of the children who participated in this study. Considering the adjusted effect size, we can say that the effect of the rGH treatment was better for LM than for the other variables assessed. There was a significant increase in total LM (kg), LMI (kg/m^2^) and LMI SDS. Several studies found a significant increase of LM, which was observed at different ages, from four months to six years of age, with a higher increase in the first year of treatment.^7,8,13,16,29^ Even in studies conducted in the early stages of puberty, LM (kg) increased significantly in all years of treatment.^7,8,27^

However, normalization of LM for length (LMI) is still little explored in similar studies. Normalization allows to evaluate separately the amount of LM in relation to length, making a distinction between individuals with different statures, and can show the changes that have occurred over time. Thus, it allows a better interpretation of changes in body composition, as shown in this study, where LMI increased after 18 months of rGH treatment.^30,31^

A gradual and functional increase of LM is fundamental for bone gain in growing children, because LM is a strong predictor of Bone Mineral Density (BMD) in childhood. Both muscle and bone are directly related and perform not only mechanical functions but also act in the secretion of trophic hormones and growth factors.^32,33^

However, the relationship of LM with the risk of future diseases is still little studied, and information about the implications of low LM in the risk of diseases in adulthood is scarce. In both sexes, the low amount of LM was associated with higher cardiovascular risks and more chances of developing diabetes mellitus type 2 and metabolic syndrome. It has been demonstrated that as LM increases there is a gradual reduction of risk factors, and that high levels of muscle fitness are inversely associated with obesity, insulin resistance, cardiovascular risk and inflammation.^33–36^

In short-stature children born SGA, the differences between both sexes show a higher percentage of LM (kg) and a lower percentage of total and abdominal FM in boys, who exhibited a higher increase of LM (kg) and a decrease of FM (kg) than girls after treatment.^7,8^ Furthermore, children born SGA at term have more LM (kg) gain compared with preterm children born SGA.^9^ In our study, all children were born at term, and a comparison between the sexes was not possible due to heterogeneity in the number of boys and girls.

The BMI/A was an indicator that exhibited no significant change over the 18-month treatment, and the indices in both periods represent normal body composition. These findings corroborate the ones of other studies, where this indicator did not show a significant alteration during the treatment with rGH.^8,10^

With respect to FM, most of the indicators assessed did not show a significant difference after 18 months of treatment, but if we consider the absolute value (kg), there is a significant increase, which can be the result of the growth and development process observed in these children. As already reported in previous studies, a progressive, but not significant increase of FM was observed in children born SGA during the rGH treatment.^8,29,37^

When this indicator (FM) was standardized for age and sex (SDS), no significant changes were observed, but different scenarios are described in the literature, showing a significant increase of FM SDS among adolescents that began this treatment at the early stages of puberty^17^ and a significant reduction of this indictor in a group of younger children born SGA, with mean age of 5.9 ±1.6 years.^12^

When examining BF%, the results presented here do not indicate significant changes, but there are studies that found a reduction of BF% after treatment with rGH.^7,8,11,16^ Nonetheless, studies report very divergent alterations of FM indicators, as a result of the rGH treatment, and not well established, because usually there is an increased or unaltered FM and reduced BF%, thus suggesting that a BF% decrease may be due to growth and not exclusively to a reduction of total FM (kg).^10^

Anyway, the benefits of rGH treatment in the body composition of children born SGA can be seen when they are compared with other children who did not receive the treatment, where the former had less total FM (kg) gain, with a reduction of BF%, than those untreated.^29^ It is also worth noting that a decrease of BF% is more notable in children who begin the treatment younger and in the ones who achieved more length gain during the treatment.^11^

When treatment began later (around 11 years old) BF% SDS was higher than the average for the reference population, staying unaltered during the treatment and significantly higher than peers of the same age and sex.^27^

Some studies present the results of distribution of body fat indicating a progressive reduction of AF in children born SGA treated with rGH, but without statistically significant difference.^7,13^ On the other hand, visceral fat increased during the treatment, but remained lower than the reference for the same age and sex.^10,13^ Such redistribution of body fat, with more fat distribution in the trunk region, does not differ between children born SGA treated with rGH and those not treated. In addition, treatment with rGH does not produce unfavorable effects on the adiposity of these children, since in some cases it can only change the distribution of body fat.^10,11^

Regarding the metabolic profile, it is typical of children born SGA to have IGF-1 levels below the ones born AGA. Children born SGA exhibit abnormal lipid profile, with cholesterol levels above the recommended values. However, treatment with rGH causes a progressive increase of IGF-1 levels and reduce total cholesterol levels, without changing significantly the levels of HDL-cholesterol and triglycerides. Alterations in blood sugar levels are usually small, with a slight increase during treatment but remaining within the reference limits.^38^

These characteristics were observed in the present study and in other research studies, where there was a significant increase of IGF-1 levels^10,12,13,16,39,40^ and glycemia, which remained below the maximum tolerance limit.^13,16,40^ The lipid profile exhibited no alterations in absolute numbers,^7,16^ but high cholesterol levels were observed in the pre-treatment period. There was a decreased number of children with altered cholesterol after the 18-month treatment.

The main limitations of this study were the small number of participants, which was a consequence of the suspension of care services and pedagogical activities during the period of COVID-19, and for not been a randomized study, with the lack of a comparable group of children, for instance, SGA children, with the same inclusion criteria, whose parents refused the rGH treatment.

On the other hand, despite the small number of participants, the results presented here are similar to the ones already described in other studies in the literature. Also, the conduction of a clinical trial, despite not being randomized, has the advantages of being a systematized study that delivers important results to science and society, in addition to providing treatment with rGH to all short children born SGA.

In conclusion, the rGH treatment provided an increase of length SDS, increased LM, LMI, and LMI SDS, with no increase of BF% and AF, as well as an improvement in lipid profile, with no changes in blood sugar levels, in children born SGA.

## Data Availability

The database that originated the article is available with the corresponding author.
