# Supplementary material for: Effect of treatment with growth hormone on body composition and metabolic profile of short children born small for gestational age
Source: Rev Paul Pediatr. 2024 Feb 12;42:e2023073. doi: 10.1590/1984-0462/2024/42/2023073 (PMC10868514; doi:10.1590/1984-0462/2024/42/2023073)
Supplement: Supplementary file 1 [file 1984-0462-rpp-42-e2023073-suppl1.docx]

**ARTIGO ORIGINAL**

**DOI:** <https://doi.org/10.1590/1984-0462/2024/42/2023073>

**ELOCATOR:** e2023073

**RECEBIDO: 10/04/2023**

**APROVADO: 10/10/2023**

**Effect of treatment with growth hormone on body composition and metabolic profile of short children born small for gestational age**

*Growth hormone and short children born small for gestational age*

**Efeito do tratamento com Hormônio de Crescimento na composição corporal e no perfil metabólico de crianças nascidas Pequenas para a Idade Gestacional com baixa estatura**

*Hormônio do crescimento e crianças nascidas pequenas para a idade gestacional*

**Adriana Masiero Kühl (**ORCID <http://orcid.org/0000-0002-9907-0169>), Universidade Estadual do Centr-Oeste, Guarapuava, Parana, Brazil.

**Márcia Regina Messaggi Gomes Dias (**ORCID <http://orcid.org/0000-0001-6158-2014>), Universidade Federal do Paraná, Curitiba, Parana, Brazil

**Rosana Marques Pereira (**ORCID <https://orcid.org/0000-0002-0270-1519>), Universidade Federal do Paraná, Curitiba, Parana, Brazil

**Author´s contribuitions**

Study design: Adriana M. Kühl, Márcia R. M. Gomes Dias and Rosana M. Pereira

Data collection: Adriana M. Kühl

Data analysis: Adriana M. Kühl, Márcia R. M. Gomes Dias and Rosana M. Pereira

Manuscript writing: Adriana M. Kühl

Manuscript revision: Márcia R. M. Gomes Dias and Rosana M. Pereira

Study supervision: Márcia R. M. Gomes Dias and Rosana M. Pereira

**Declaration:** the database that originated the article is available upon request, with a corresponding author.

**Corresponding author:** Name: Adriana Masiero Kühl

Adress: 1993Professora Amálio Pinheiro St, Guarapuava, Parana, 85015334, Brazil.

Phone: (42) 99977-6444. e-mail: amasiero@unicentro.br

**Conflict of interests**

The authors declare there is no conflict of interests.

**Funding**

The study did not receive any funding.

**Total number of words:** text 3136; abstract 224; references 42; tables 4; figures 0.

**ABSTRACT**

**Objective:** To assess the effect of recombinant Growth Hormone (rGH) on body composition and metabolic profile of prepubertal short children born Small for Gestational Age (SGA) before and after 18 months of treatment.

**Methods**: It is a clinical, non-randomized, and paired study. Children born SGA, with birth weight and/or lenght <-2SD for gestational age and sex, prepubertal, born at full term, of both genders, with the indication for treatment with rGH were included. The intervention was performed with biosynthetic rGH at doses ranging from 0.03 to 0.05 mg/kg/day, administered subcutaneously, once a day at bedtime. Total lean mass (LM) and total fat mass (FM) were carried out using dual-energy X-ray absorptiometry (DXA), and the metabolic profile was assessed for insulin, glycemia, IGF-1 levels and lipid profile.

**Results:** 12 patients (9 girls, 8.17±2.39y) were evaluated; 3 patients dropped out of the study. There was an increase of LM adjusted for lenght (LMI) (p=0.008), LMI SDS adjusted for age and sex (p=0.007), and total LM (p<0.001). The percentage of body fat (BF%) and abdominal fat (AF) remained unaltered in relation to the beginning of treatment. Among the metabolic variables, blood glucose remained within normal levels, and there was a reduction in the number of participants with altered cholesterol (p=0.023).

**Conclusions**: The effect of rGH treatment was higher on LM than in FM, with increased LM adjusted for lenght and standardized for age and sex. Glycemia remained within the normal limits, and there was a decreased number of children with total cholesterol above the recommended levels.

**Keywords**: Infant, Small for Gestational Age, Body Composition, Recombinant Growth Hormone

**RESUMO**

**Objetivo**: avaliar o efeito do Hormônio de Crescimento Recombinante (rHC) na composição corporal e no perfil metabólico de crianças pré-púberes com baixa estatura, nascidas pequenas para a Idade Gestacional (PIG) antes e após 18 meses de tratamento.

**Métodos**: Eestudo clínico, não randomizado e pareado. Foram incluídas crianças nascidas PIG, com peso e/ou altura ao nascer <-2DP para idade gestacional e sexo, pré-púberes, nascidas a termo, de ambos os sexos, com indicação de tratamento com rGH. A intervenção foi realizada com rGH biossintético com doses variando de 0,03 a 0,05 mg/kg/dia, administrado por via subcutânea, uma vez ao dia ao deitar-se. A massa magra total (LM) e a massa gorda total (MG) foram determinadas por meio de absorciometria de raios X de dupla energia (DXA), e o perfil metabólico foi avaliado com dosagens de insulina, glicemia, IGF-1 e perfil lipídico.

**Resultados**: 12 pacientes (9 meninas, 8,17±2,39 anos) foram avaliados; 3 pacientes abandonaram o estudo. Houve aumento da LM ajustada para estatura (LMI) (p=0,008), LMI SDS ajustada para idade e sexo (p=0,007) e LM total (p<0,001). O percentual de gordura corporal (GC%) e gordura abdominal (AF) permaneceram inalterados em relação ao início do tratamento. Dentre as variáveis metabólicas, a glicemia manteve-se dentro da normalidade, e houve redução do número de participantes com colesterol alterado (p=0,023).

**Conclusões**: O efeito do tratamento com HCr foi maior na MM do que na MG, com o aumento da MM ajustada para altura e padronizada para idade e sexo. A glicemia permaneceu normal e houve redução do número de crianças com colesterol total acima do recomendado.

**Palavras chaves**: Recém-Nascido Pequeno para a Idade Gestacional, Composição Corporal, Hormônio do Crescimento Recombinante

**INTRODUCTION**

Childhood is a period of fast growth and development, highly vulnerable to health complications in the presence of unfavorable conditions. Newborns with weight and/or length below -2 standard deviation scores (SDS) for gestational age and sex are classified as small for the gestational age (SGA) and are more susceptible to health problems in the neonatal period and development of chronic diseases in adulthood. The causes for SGA are diverse and can be associated with intrauterine growth restriction, ethnicity, or short maternal stature.^1-3^

Most children born SGA show spontaneous catch-up growth into normal lenght range (> −2 SDS) during childhood, but about 10% remain short after four years old and exhibit less lean mass (LM). In turn, when there is a fast and increased body weight during the catch-up growth period, these children may exhibit higher fat mass (FM), with deposition of fat in the central region of the body, higher blood glucose and body pressure levels.^4,5^

Recombinant growth hormone (rGH) treatment has shown good tolerance, low incidence of adverse effects and positive results in increasing stature, in improving lipid metabolism and regularizing blood pressure and metabolic factors. rGH also changes body composition of these children, contributing to increasing LM or maintaining or diminishing FM.^4,6^

This treatment is recommended for children born SGA with a lenght <-2SD and are at least two years old. The treatment should preferably begin in prepuberty, with doses ranging from 0.03 and 0.07 mg/kg/day and can last until the children reach adult length.^7-13^

Body composition in most of these studies was assessed by dual-energy X-ray absorptiometry (DXA), which is considered the gold standard for assessment of body mass composition, especially FM. This method has good accuracy, is fast, painless, and the child can stay awake during the exam. The DXA execution method is safe, using low-intensity X-rays, which allows to measure bone mass, LM and FM individually.^14,15^

Some studies show a significant increase in LM throughout the treatment, at the end of the follow-up period, and in comparison with the control group, with the effect being more evident in boys and during the first year of treatment. The main determinant of reported LM gain is lenght, as growth recovery was accompanied by an increase in LM, especially during the first year of treatment. It's noteworthy that children who underwent the treatment gained less FM than those who were untreated.^7,13,39,27^

Therefore, it can be assumed that children born SGA, when subjected to growth hormone therapy, experience an improvement in growth and body composition. This study aimed to assess the effect of rGH treatment on body composition and metabolic health of short children born SGA before and after 18 months of treatment.

**METHOD**

It is a clinical, non-randomized, paired study carried out with short children born SGA who received treatment with rGH at the Pediatric Endocrinology Unit of the Clinical Hospital Complex at the Federal University of Paraná (UEP/CHC/UFPR) from Oct 2018 to Dec 2021.

Elegibility criteria included prepubertal short children of both sexes born SGA,^16^ with weight and/or lenght at birth <-2SD for gestational age and sex,^17^ born at full term, with indication for treatment with rGH and whose parents and/or guardians agreed to participate in the study and signed the Free and Informed Consent Form in writing.

Children born preterm, with bone dysplasia, genetic syndromes, deficiency of growth hormone and other hormones, and children already in puberty at the beginning of the treatment were excluded from the study.

Twelve patients were included in the study and evaluated in 2 moments: immediately before starting treatment with rGH and 18 months later. Three children were lost to follow-up, resulting in nine children evaluated at the end of the study. Losses during the study comprised individuals who abandoned treatment (n=1) and conclusion of data collection before completing 18 months of treatment (n=2).

All children were followed up by pediatric endocrinologists and received treatment with biosynthetic rGH with doses ranging from 0.03 to 0.05 mg/kg/day, administered subcutaneously, once a day at bedtime.

Body mass composition and metabolic variables were assessed prior to the beginning of the treatment (4.0±1.7 months before) and 18 months afterwards. All data were collected by one single researcher in both periods.

Body composition was assessed by Dual-Energy X-Ray Absorptiometry (DXA) at the Bone Densitometry Sector of the Clinical Hospital Complex of UFPR (CHC-UFPR), in a Lunar Prodigy Advance® whole-body scanner (GE Medical Systems, Madison, WI, USA) and Encore® software. All assessments were carried out using the same equipment, by the same person, with calibration performed every day in the morning prior to the first scan.

During the scans, the patients wore light clothes without metallic parts and were asked to remove all metallic objects. They were placed in supine position with the body centralized at the scanning table, with the feet in neutral position and secured with a Velcro strip, hands facing downwards, 1cm away from the body, laying still as proposed by the NHANES’s protocol.^20^

Based on the DXA examination, FM values, percentage of body fat (BF%) and LM were determined. With absolute FM and LM values, the Lean Mass Index (LMI) [total lean mass (kg)/length (m2)] and Fat Mass Index (FMI) [total fat mass (kg)/lenght (m2)] were also calculated. All these values, FM, BF%, LM, LMI and FMI were transformed into Standard Deviation Scores (SDS) standardized for sex and age, based on two Brazilian reference populations, one for children under 10 years of age^21^ and the other for those over 1 year.^22^

DXA also enables to assess regional distribution of body fat by using a standard configuration for segmental analysis, based on which the total abdominal fat (AF) and the percentage of abdominal fat (AF%) could be determined. This region, also called android region, comprises the area located between the ribs and pelvis, with demarcation greater than 20% of the distance between the iliac crest and the neck and a lower demarcation at the top of the pelvis.^14^

The clinical variables related to weight and length at birth, gestational age (GA) and pubertal stage were obtained based on a research protocol for data collection from medical records developed by UEP/CHC/UFPR. Current weight and lenght were measured using a digital scale (Filizola®) and fixed stadiometer (Stadiometer Mode S100®), respectively, and measurements were performed following the recommendations of the Ministry of Health.^23^ To assess nutritional status, the anthropometric indices of body mass index for age (BMI/A) and stature for age (S/A) were determined according to WHO’s recommendations and cutoff scores.^16^

The evaluation of the metabolic parameters was carried out at the Clinical Analysis Laboratory of the Clinical Hospital (ULAC/CHC/UFPR), with blood collected by vein puncture after 12 hours of fasting. Glucose, insulin, total cholesterol, HDL-cholesterol and triglycerides levels were measured using the Anility equipment, with specific kits for each component.

Lipid and insulin profiles were evaluated according to the *I Diretriz de Prevenção da Aterosclerose na Infância e na Adolescência*^24^ [1^st^ Guideline for the Prevention of Atherosclerosis in Childhood and Adolescence], and the desirable levels considered were: for total cholesterol <150 mg/dL; for triglycerides and LDL-cholesterol (LDL-COL) <100 mg/dL; for HDL-cholesterol (HDL-COL) >45 mg/dL and insulin <15 mUI/L. For fasting glucose, a glucose level of <100 mg/dL was considered adequate.^25^

Insulin resistance (IR) was determined by the Homeostasis Model Assessment for Insulin Resistance (HOMA-IR) index using the HOMA2 Calculator® software, which uses fasting glucose added by insulin in the formulation. HOMA-IR values over 2.5 units were defined.^26^

This study was approved by the Human Research Ethics Committee from CHC-UFPR with registration no. 94100318.4.0000.0096. All participants received the Informed Consent Form and for those aged ten years or over, the Informed Assent Term was administered.

Data homogeneity was assessed using the Kolmogorov-Smirnov and Shapiro-Wilk normality tests, which showed normal distribution for most variables. Mean () and Standard Deviation (SD) were used for descriptive statistics. For comparison of the means before and after 18 months of treatment, Student's t-test was used for dependent samples, for variables with normal distribution. For variables that did not present normal distribution, the Wilcoxon test was used. The categorical variables were assessed by the McNemar test, and when not possible, the Chi-Square test was used. Significance level was determined as p<0.05. The analyses were carried out using the SPSS statistical software (version 20).

The effect size was calculated by the Cohen’s d test, using the GPower 3.1.9.7 software, and values ≥0.8 were assumed as having large effect size; between 0.8 and 0.2 they were considered as medium effect size, and <0.2 as small effect size.

**RESULTS**

Nine children participated in the study and were evaluated in pre-treatment period and after 18 months of treatment with rGH. All children were in the prepubertal period before the treatment, the initial mean age was 9,0±2.7, and final age was 10.7±2.7. The majority were female (55%), born at term (GA≥38 weeks) and with length <-2 SD (Table 1). With respect to the anthropometric characteristics, only lenght (p<0.001) increased. Weight, BMI/A, and waist circumference stayed unaltered and within the normal levels (Table 2).

The results of assessment of body composition show that after 18 months of treatment with rGH, there was an increase in LM (kg) from 17.1±5.9kg to 22.7±7.5kg (p<0.001), as well as in LM adjusted for lenght (LMI) and LMI SDS adjusted for age and sex. There was no change in FM when the same adjustments were used, as well as for BF%, AF and AF%, which remained unaltered (Table 3).

The treatment with rGH had better effect on LM than on the other variables (*d*=0.81) with an increase of 5.6kg after 18 months of treatment (Table 3).

Table 4 contains the results of the metabolic evaluation, where it can be seen that the children started the treatment with total cholesterol levels above the recommended values (165.0±21.3mg/dL), and there was an improvement after 18 months of treatment. It was found an increase of IGF-1 levels (188.8±48.2 to 403.1±116.4mg/ml), and the glucose levels remained within the recommended levels for age.

With respect to the lipid profile, only total cholesterol exhibited a statistical difference between the assessments, showing a decrease in the number of participants with inadequate cholesterol levels from 75% (n=9) to 66.7% (n=6) (p=0.023).

**DISCUSSION**

This study showed longitudinal results of 18 months of treatment with rGH in body composition assessed by DXA and in the metabolic profiles of short children born SGA. Children born SGA exhibited BMI/ (z-score), LM (SDS), BF% (SDS) and FM (SDS) lower than the mean value for the population of the same age and sex at the beginning of the treatment, with an increase in total LM (Kg), LMI and LMI SDS after the treatment period. There was an increase in the lipid profile, with a decreased number of children with altered cholesterol.

A study in the literature reports a profile similar to the one found here, where short-stature children born SGA usually exhibit lower BMI/A, combined with a lower amount of LM and FM, than children of the same age and sex born with adequate weight and/or stature for the gestational age.^7,10,11,13,27,28^ This condition may be the effect of a failure in catching-up growth in early childhood, when about 10% of the children born SGA remain smaller than other children of the same age and sex, causing children born SGA to have an average lenght score lower than the reference population.^29^

For these children, rGH treatment has been proven to be safe and with positive results both for lenght catch-up and for lipid profile and body composition, especially when this treatment starts during prepuberty.^4,6^ In this study, all children were at the prepubertal phase when the treatment began and succeeded in achieving catch-up in lenght, better levels of total cholesterol and increased LM.

LM increase was the major alteration found in the body composition of the children who participated in this study. Considering the adjusted effect size, we can say that the effect of the rGH treatment was better for LM than for the other variables assessed. There was a significant increase in total LM (kg), LMI (kg/m^2^) and LMI SDS. Several studies found a significant increase of LM, which was observed at different ages, from four months to six years of age, with a higher increase in the first year of treatment.^7,8,13,27,30^ Even in studies conducted in the early stages of puberty, LM (kg) increased significantly in all years of treatment.^7,8,28^

However, normalization of LM for lenght (LMI) is still little explored in similar studies. Normalization allows to evaluate separately the amount of LM in relation to lenght, making a distinction between individuals with different statures, and can show the changes that have occurred over time. Thus, it allows a better interpretation of changes in body composition, as shown in this study, where LMI increased after 18 months of rGH treatment.^31,32^

A gradual and functional increase of LM is fundamental for bone gain in growing children, because LM is a strong predictor of Bone Mineral Density (BMD) in childhood. Both muscle and bone are directly related and perform not only mechanical functions but also act in the secretion of trophic hormones and growth factors.^33,34^

However, the relationship of LM with the risk of future diseases in still little studied, and information about the implications of low LM in the risk of diseases in adulthood is scarce. In both sexes, the low amount of LM was associated with higher cardiovascular risks and more chances of developing diabetes mellitus type 2 and metabolic syndrome. It has been demonstrated that as LM increases there is a gradual reduction of risk factors, and that high levels of muscle fitness are inversely associated with obesity, insulin resistance, cardiovascular risk and inflammation.^35-37^

In short-stature children born SGA, the differences between both sexes show a higher percentage of LM (kg) and a lower percentage of total and abdominal FM in boys, who exhibited a higher increase of LM (kg) and a decrease of FM (kg) than girls after treatment.^7,8^ Furthermore, children born SGA at term have more LM (kg) gain compared with preterm children born SGA.^9^ In our study, all children were born at term, and a comparison between the sexes was not possible due to heterogeneity in the number of boys and girls.

The BMI/A was an indicator that exhibited no significant change over the 18-month treatment, and the indices in both periods represent normal body composition. These findings corroborate the ones of other studies, where this indicator did not show a significant alteration during the treatment with rGH.^8,10^

With respect to FM, most of the indicators assessed did not show a significant difference after 18 months of treatment, but if we consider the absolute value (kg), there is a significant increase, which can be the result of the growth and development process observed in these children. As already reported in previous studies, a progressive, but not significant increase of FM was observed in children born SGA during the rGH treatment.^8,30,38^

When this indicator (FM) was standardized for age and sex (SDS), no significant changes were observed, but different scenarios are described in the literature, showing a significant increase of FM SDS among adolescents that began this treatment at the early stages of puberty^39^ and a significant reduction of this indictor in a group of younger children born SGA, with mean age of 5.9 ±1.6 years.^12^

When examining BF%, the results presented here do not indicate significant changes, but there are studies that found a reduction of BF% after treatment with rGH.^7,8,11,27^ Nonetheless, studies report very divergent alterations of FM indicators, as a result of the rGH treatment, and not well established, because usually there is an increased or unaltered FM and reduced BF%, thus suggesting that a BF% decrease may be due to growth and not exclusively to a reduction of total FM (Kg).^10^

Anyway, the benefits of rGH treatment in the body composition of children born SGA can be seen when they are compared with other children who did not receive the treatment, where the former had less total FM (kg) gain, with a reduction of BF%, than those untreated.^30^ It is also worth noting that a decrease of BF% is more notable in children who begin the treatment younger and in the ones who achieved more lenght gain during the treatment.^11^

When treatment begins later (around 11 years old) BF% SDS was higher than the average for the reference population, staying unaltered during the treatment and significantly higher than peers of the same age and sex.^28^

Some studies present the results of distribution of body fat indicating a progressive reduction of AF in children born SGA treated with rGH, but without statistically significant difference.^7,13^ On the other hand, visceral fat increased during the treatment, but remained lower than the reference for the same age and sex.^10,13^ Such redistribution of body fat, with more fat distribution in the trunk region, does not differ between children born SGA treated with rGH and those not treated. In addition, treatment with rGH does not produce unfavorable effects on the adiposity of these children, since in some cases it can only change the distribution of body fat.^10,11^

Regarding the metabolic profile, it is typical of children born SGA to have IGF-1 levels below the ones born AGA. Children born SGA exhibit abnormal lipid profile, with cholesterol levels above the recommended values. However, treatment with rGH causes a progressive increase of IGF-1 levels and reduce total cholesterol levels, without changing significantly the levels of HDL-cholesterol and triglycerides. Alterations in blood sugar levels are usually small with a slight increase during treatment but remaining within the reference limits.^40^

These characteristics were observed in the present study and in other research studies, where there was a significant increase of IGF-1 levels^10,12,13,27,41,42^ and glycemia, which remained below the maximum tolerance limit.^13,27,41^ The lipid profile exhibited no alterations in absolute numbers,^7,27^ but high cholesterol levels were observed in the pre-treatment period. There was a decreased number of children with altered cholesterol after the 18-month treatment.

The main limitations of this study were the small number of participants, which was a consequence of the suspension of care services and pedagogical activities during the period of COVID-19, and for not been a randomized study, with the lack of a comparable group of children, for instance, SGA children, with the same inclusion criteria, whose parents refused the rGH treatment.

On the other hand, despite the small number of participants, the results presented here are similar to the ones already described in other studies in the literature. Also, the conduction of a clinical trial, despite not being randomized, has the advantages of being a systematized study that delivers important results to science and society, in addition to providing treatment with rGH to all short children born SGA.

In conclusion, the rGH treatment provided an increase of lenght SDS, increased LM, LMI, and LMI SDS, with no increase of BF% and AF, as well as an improvement in lipid profile, with no changes in blood sugar levels, in children born SGA.

**Data Availability:** original data generated and analyzed during this study are included in this published article

**REFERENCES**

1. Lee PA, Chernausek SD, Hokken-Koelega AC, Czernichow P. International small for gestational age advisory board consensus development conference statement: management of short children born small for gestational age. Pediatrics. 2003;111:1253-61. https://doi.org/10.1542/peds.111.6.1253

2. Francis A, Hugh O, Gardosi J. Customized vs INTERGROWTH-21 st standards for the assessment of birthweight and stillbirth risk at term. Am J Obstet Gynecol. 2018;218(Suppl 2): S692-9. https://doi.org/10.1016/j.ajog.2017.12.013

3. Falcão IR, Ribeiro-Silva RD, Almeida MF, Fiaccone RL, Silva NJ, Paixao ES, et al. Factors associated with small- And large-for-gestational-age in socioeconomically vulnerable individuals in the 100 million Brazilian Cohort. Am J Clin Nutr. 2021;114:109-16. https://doi.org/10.1186/s12884-020-03226-x

4. Van der Steen M, Hokken-Koelega AC. Consequences of being born small for gestational age. Pediatr Adolesc Med. 2020;22:43-58. https://doi.org/10.1159/000495433

5. Marcovecchio ML, Gorman S, Watson LP, Dunger DB, Beardsall K. Catch-up growth in children born small for gestational age related to body composition and metabolic risk at six years of age in the UK. Horm Res Paediatr. 2020; 93:119-27. https://doi.org/10.1159/000508974

6. Hwang IT. Efficacy and safety of growth hormone treatment for children born small for gestational age. Korean J Pediatr. 2014; 57:379-83. https://doi.org/10.3345/kjp.2014.57.9.379

7. Clemente EA, Villagrasa PS, Casas AA, Frontera PR, Aznar LA, Lozano GB. Composición corporal y riesgo metabólico en niños pequeños para la edad gestacional en tratamiento con hormona del crecimiento. Med Clin. 2016;147:231-7. https://doi.org/10.1016/j.medcli.2016.06.002

8. Clemente EA, Villagrasa PS, Casas AA, Frontera PR, Lozano GB, Aznar LA, et al. Modificaciones en variables antropométricas, analíticas de riesgo metabólico y composición corporal en pequeños para la edad gestacional en tratamiento con hormona de crecimiento. An Pediatr. 2017; 86:240-8. https://doi.org/10.1016/j.anpedi.2016.05.001

9. Kort SW, Willemsen RH, Van der Kaay DC, Hokken-Koelega AC. The effect of growth hormone treatment on metabolic and cardiovascular risk factors is similar in preterm and term short, small for gestational age children. Clin Endocrinol. 2009;71:65-73. doi:10.1111/j.1365-2265.2008.03504.x

10. Maeyama T, Ida S, Onuma S, Shoji Y, Yamamoto T, Etani Y, et al. Fat distribution in short-stature children born small for gestational age. Pediatr Int. 2020;62:1351-6. https://doi.org/10.1111/ped.14337

11. Willemsen RH, Arends NJ, Waarde WM, Jansen M, van Mil EG, Mulder J, et al. Long-term effects of growth hormone (GH) treatment on body composition and bone mineral density in short children born small-for-gestational-age: six-year follow-up of a randomized controlled GH trial. Clin Endocrinol. 2007;67:485-92. https://doi.org/10.1111/j.1365-2265.2007.02913.x

12. Boonstra VH, Arends NJ, Stijnen T, Blum WF, Akkerman O, Hokken-Koelega AC. Food intake of children with short stature born small for gestational age before and during a randomized GH trial. Horm Res. 2006;65:23-30. https://doi.org/10.1159/000090376

13. Ibáñez L, Lopez-Bermejo A, Díaz M, Jaramillo A, Marín S, Zegher F. Growth hormone therapy in short children born small for gestational age: effects on abdominal fat partitioning and circulating follistatin and high-molecular-weight adiponectin. J Clin Endocr Metab. 2010;95:2234-9. https://doi.org/10.1210/jc.2009-2805

14. Maeda SS, Albergaria BH, Szejnfeld VL, Castro ML, Arantes HP, Ushida M, et al. Official Position of the Brazilian Association of Bone Assessment and Metabolism (ABRASSO) on the evaluation of body composition by densitometry - part II (clinical aspects): interpretation, reporting, and special situations. Adv Rheumatol. 2022;62:11. https://doi.org/10.1186/s42358-022-00240-9

15. Cieśluk K, Dobroch J, Sawicka-Żukowska M, Krawczuk-Rybak M. Body composition measurements in paediatrics - a review. Part 2. Pediatr Endocrinol Diabetes Metab. 2018;24:191-6. https://doi.org/10.5114/pedm.2018.83366

16. World Health Organization. WHO child growth standards: methods and development: length/height-for-age, weight-for-age, weight-for-length, weight-for-height and body mass index-for-age. WHO; 2006.

17. Villar J, Ismail LC, Victora CG, Ohuma EO, Bertino E, Altman DG, et al. International standards for newborn weight, length, and head circumference by gestational age and sex: the newborn cross-sectional study of the INTERGROWTH-21st Project. Lancet. 2014;384:857-68. https://doi.org/10.1016/S0140-6736(14)60932-6

18. Marshall WA, Tanner JM. Variations in the pattern of pubertal changes in boys. Arch Dis Child. 1970;45:13-23. http://dx.doi.org/10.1136/adc.45.239.13

19. Marshall WA, Tanner JM. Variations in pattern of pubertal changes in girls. Arch Dis Child. 1969;44:291-303. http://dx.doi.org/10.1136/adc.44.235.291

20. Center for Health Statistics (CDC). Body composition procedures manual. National Health and Nutrition Examination Survey (NHANES); 2013.

21. Zanini RV, Santos IS, Gigante DP, Matijasevich A, Barros FC, Barros AJ. Body composition assessment using DXA in six-year-old children: the 2004 Pelotas Birth Cohort, Rio Grande do Sul State, Brazil. Cad Saude Publica. 2014;30:2123-33. https://doi.org/10.1590/0102-311X00153313

22. Ripka WL, Orsso CE, Haqq AM, Luz TG, Prado CM, Ulbricht L. Lean mass reference curves in adolescents using dual-energy x-ray absorptiometry (DXA). PLoS One. 2020;15:1-11. https://doi.org/10.1371/journal.pone.0228646

23. Brazil. Ministério da Saúde. Orientações para a coleta e análise de dados antropométricos em serviços de saúde: norma técnica do sistema de vigilância alimentar e nutricional - SISVAN. Ministério da Saúde. Secretaria de Atenção à Saúde. Departamento de Atenção Básica; 2011.

24. Giuliano IC, Caramelli B, Pellanda L, Duncan B, Mattos S, Fonseca FH. I Diretriz de prevenção da aterosclerose na infância e na adolescência. Arq Bras Cardiol. 2005;85(Suppl VI):S3-36.

25. Weffort VR, Maranhão HS, Mello ED, Barretto JR, Fisberg M, Moretzsohn MA, et al. Manual de avaliação nutricional - vol 2. Sociedade Brasileira de Pediatria; 2021.

26. d’Annunzio G, Vanelli M, Meschi F, Pistorio A, Caso M, Pongigline C, et al. Valori normali di HOMA-IR in bambini e adolescenti: studio multicentrico italaiano. Quad Pediatr. 2004;3:44.

27. Thankamony A, Jensen RB, O'Connell SM, Day F, Kirk J, Donaldson M, et al. Adiposity in children born small for gestational age is associated with β-cell function, genetic variants for insulin resistance, and response to growth hormone treatment. J Clin Endocr Metab. 2016;101:131-42. https://doi.org/10.1210/jc.2015-3019

28. Steen M, Lem AJ, Kaay DC, Waarde WM, Hulst FJ, Neijens FS, et al. Metabolic health in short children born small for gestational age treated with growth hormone and gonadotropin-releasing hormone analog: results of a randomized, dose-response trial. J Clin Endocr Metab. 2015;100:3725-34. https://doi.org/10.1210/jc.2015-2619

29. Tamaro G, Pizzul M, Gaeta G, Servello R, Trevisan M, Böhm P, et al. Prevalence of children born small for gestational age with short stature who qualify for growth hormone treatment. Ital J Pediatr. 2021;47. https://doi.org/10.1186/s13052-021-01026-3

30. Schepper J, Thomas M, Beckers D, Craen M, Maes M, Zegher F. Growth hormone treatment and fat redistribution in children born small for gestational age. J Pediatr. 2008;152:327-30. https://doi.org/10.1016/j.jpeds.2007.07.043

31. Wells JC, Cole TJ. Adjustment of fat-free mass and fat mass for height in children aged 8 y. Int J Obes. 2002;26:947-52. https://doi.org/10.1038/sj.ijo.0802027

32. Vanltaiie TB, Yang MU, Heymsfield SB, Funk RC, Boileau RA. Height-normalized indices of the body’s fat-free mass and fat mass: potentially useful indicators of nutritional status. Am J Clin Nutr. 1990;52:953-9. https://doi.org/10.1093/ajcn/52.6.953

33. Deodati A, Manco M, Mariani M, Bocchini S, Högler W, Cappa M, et al. Bone density and body composition in small for gestational age children with adequate catch-up growth: a preliminary retrospective case control study. Bone. 2021;153. https://doi.org/10.1016/j.bone.2021.116114

34. Nordman H, Voutilainen R, Laitinen T, Antikainen L, Jääskeläinen J. Birth size, body composition, and adrenal androgens as determinants of bone mineral density in mid-childhood. Pediatr Res. 2018; 83:993-8. https://doi.org/10.1038/pr.2018.12

35. Kim S, Valdez R. Metabolic risk factors in U.S. youth with low relative muscle mass. Obes Res Clin Pract. 2015;9:125-32. https://doi.org/10.1016/j.orcp.2014.05.002

36. Burrows R, Correa-Burrows P, Reyes M, Blanco E, Albala C, Gahagan S. High cardiometabolic risk in healthy Chilean adolescents: associations with anthropometric, biological and lifestyle factors. Public Health Nutr. 2016;19:486-93. https://doi.org/10.1017/S1368980015001585

37. Wells JC. Body composition in childhood: effects of normal growth and disease. Proc Nutr Soc. 2003;62:521-8. https://doi.org/10.1079/PNS2003261

38. Leger J, Garel C, Fjellestad-Paulsen A, Hassan M, Czernichow P. Human growth hormone treatment of short-stature children born small for gestational age: effect on muscle and adipose tissue mass during a 3-year treatment period and after 1 year’s withdrawal. J Clin Endocr Metab. 1998;83:3512-6. https://doi.org/10.1210/jcem.83.10.5165

39. Lem AJ, Kaay DC, Hokken-Koelega AC. Bone mineral density and body composition in short children born SGA during growth hormone and gonadotropin releasing hormone analog treatment. J Clin Endocr Metab. 2013;98:77-86. [https://doi.org/10.1210/jc.2012-249240.](https://doi.org/10.1210/jc.2012-249240.%20)

40. Dunger D, Darendeliler F, Kandemir N, Harris M, Rabbani A, Kappelgaard AM. What is the evidence for beneficial effects of growth hormone treatment beyond height in short children born small for gestational age? A review of published literature. J Pediatr Endocrinol Metab. 2020;33:53-70. https://doi.org/10.1515/jpem-2019-0098

41. Martin DD, Schweizer R, Schönau E, Binder G, Ranke MB. Growth hormone-induced increases in skeletal muscle mass alleviates the associated insulin resistance in short children born small for gestational age, but not with growth hormone deficiency. Horm Res. 2009;72:38-45. <https://doi.org/10.1159/000224339>

42. Berndt C, Schweizer R, Ranke MB, Binder G, Martin DD. Height, muscle, fat and boneesponse to growth hormone in short children with very low birth weight born appropriate for gestational age and small for gestational age. Horm Res Paediatr. 2014;82:81-8. https://doi.org/10.1159/000358520

**Table 1 – Initial clinical characteristics of children born SGA**

| **Variable** | ** (SD)** |
| --- | --- |
| GA (weeks) | 38.9 (0.9) |
| Weight at birth (g) | 2619 (525) |
| Weight at birth (Z-score) | -1.4 (0.9) |
| Length at birth (cm) | 44.1 (2.1) |
| Length at birth (Z-score) | -2.6 (0.6) |

GA: gestational age; SD: standard deviation

**Table 2: Changes in the anthropometric characteristics from the beginning of the rGH treatment and after 18 months**

|  | **Initial (n=9)**  ** (SD)** | **18 months (n=9)**  ** (SD)** | ***p*** |
| --- | --- | --- | --- |
| Weight (z-score) | -1,6 (1,7) | -1,1 (1,0) | 0,301 |
| Stature (z-score) | -2.8 (0.4) | -1.8 (0.5) | <0.001 |
| BMI (z-score) | -0.2 (1.2) | -0.1 (1.2) | 0.537 |
| Waist circumference (cm) | 53.7 (6.9) | 54.7 (14.7) | 0.758 |

BMI: Body Mass Index; SD: standard deviation

**Table 3 – Body composition at the beginning of the treatment with rGH and after 18 months of treatment**

|  | **Initial (n=9)**  **(SD)** | **18 months (n=9)**  **(SD)** | **P-value** | **Variation** | ***d**** |
| --- | --- | --- | --- | --- | --- |
| LM (kg) | 17.1 (5.9) | 22.8 (7.6) | <0.001 | 5.62 | 0.81 |
| LM (SDS) | -1.4 (1.4) | -0.6 (1.3) | 0.162 | - | - |
| LMI (Kg/m^2^) | 12.3 (1.5) | 13.1 (1.8) | 0.008 | 0.78 | 0.46 |
| LMI (SDS) | -0.4 (0.9) | 0.2 (1.0) | 0.007 | 0.56 | 0.58 |
| BF% | 17.6 (5.7) | 19.5 (8.2) | 0.382 | - | - |
| BF%(SDS) | -0.6 (0.8) | -0.5 (0.9) | 0.901 | - | - |
| FM (kg) | 3.8 (2.1) | 6.1 (3.9) | 0.033 | 2.34 | 0.69 |
| FM (SDS) | -0.7 (0.7) | -0.6 (0.6) | 0.426 | - | - |
| FMI (Kg/m^2^) | 2.7 (1.2) | 3.4 (1.9) | 0.130** | - | - |
| FMI (SDS) | -0.4 (0.6) | -0.1 (0.9) | 0.145 | - | - |
| AF (kg) | 0.3 (0.3) | 0.4 (0.2) | 0.960 | - | - |
| AF% | 17.0 (6.9) | 19.8 (8.3) | 0.181 | - | - |

BF%: percentage of body fat; SDS: standardized standard deviation scores; FM: total fat mass; FMI: fat mass index; AF: abdominal fat; LM: total lean mass; LMI: lean mass index.

* Adjusted effect size *(Cohen’s D) ** Wilcoxon Test*

**Table 4 –Metabolic profile at the beginning of the treatment with rGH and after 18 months of treatment**

|  | **Initial (n=9)**  **(SD)** | **18 months (n=9)**  **(SD)** | **p-value** |
| --- | --- | --- | --- |
| Insulin (mUI/dL) | 5.7(4.3) | 10.3(7.7) | 0.139* |
| HOMA-IR (units) | 0.7(0.5) | 1.3(0.9) | 0.139* |
| Glucose (mg/dL) | 77.2(6.5) | 85.0(6.9) | 0.017 |
| Total cholesterol (mg/dL) | 165.0 (21.3) | 158.3(17.2) | 0.324 |
| LDL – cholesterol (mg/dL) | 98.3(18.0) | 93.1(16.4) | 0.409 |
| HDL – cholesterol (mg/dL) | 50.9(11.1) | 47.2(8.8) | 0.336 |
| Triglycerides (mg/dL) | 72.4(34.9) | 99.8(88.3) | 0.508 |
| IGF-1 (mg/ml) | 188.8(48.2) | 403.1(116.4) | 0.001 |

SD: standard deviation * Teste de Wilcoxon
